# Practical tips for teaching academic integrity in the digital age

**DOI:** 10.15694/mep.2019.000142.1

**Published:** 2019-06-21

**Authors:** Jenan Younis, Faye Gishen

**Affiliations:** 1 UCL Medical School; 2 Royal Free Hospital

**Keywords:** Academic digital integrity, cheating, undergraduate, medical school

## Abstract

This article was migrated. The article was marked as recommended.

**Background:** Medical education, as with other areas of healthcare education, is susceptible to cheating, with national and international examples cited in the literature. There are documented examples in the lay press, but limited scholarly activity in the field, which can be a challenging area to research and tackle. We have begun to explicitly address academic integrity within our undergraduate curriculum, including a focus on plagiarism, self-plagiarism, and covert sharing of questions. We believe this is an important curricular topic as exhibiting unprofessional behaviours can correlate with professional practice and can potentially have implications for practitioners and patients.

**Aim:** To present 12 tips on teaching academic integrity in the digital age to medical students.

**Method:** The tips presented are based on our experiences of teaching academic digital integrity to medical students, primarily in the form of a scenario based quiz. We do also extrapolate from content on academic integrity elsewhere within our professionalism syllabus.

**Results:** The tips suggest that early, contemporary and contextualised material that is co-produced with students may offer a useful prophylactic approach to teaching about academic integrity.

**Conclusions:** The principles presented could be adapted to other healthcare students and settings, including postgraduate education.

## Introduction

**Figure 1.**
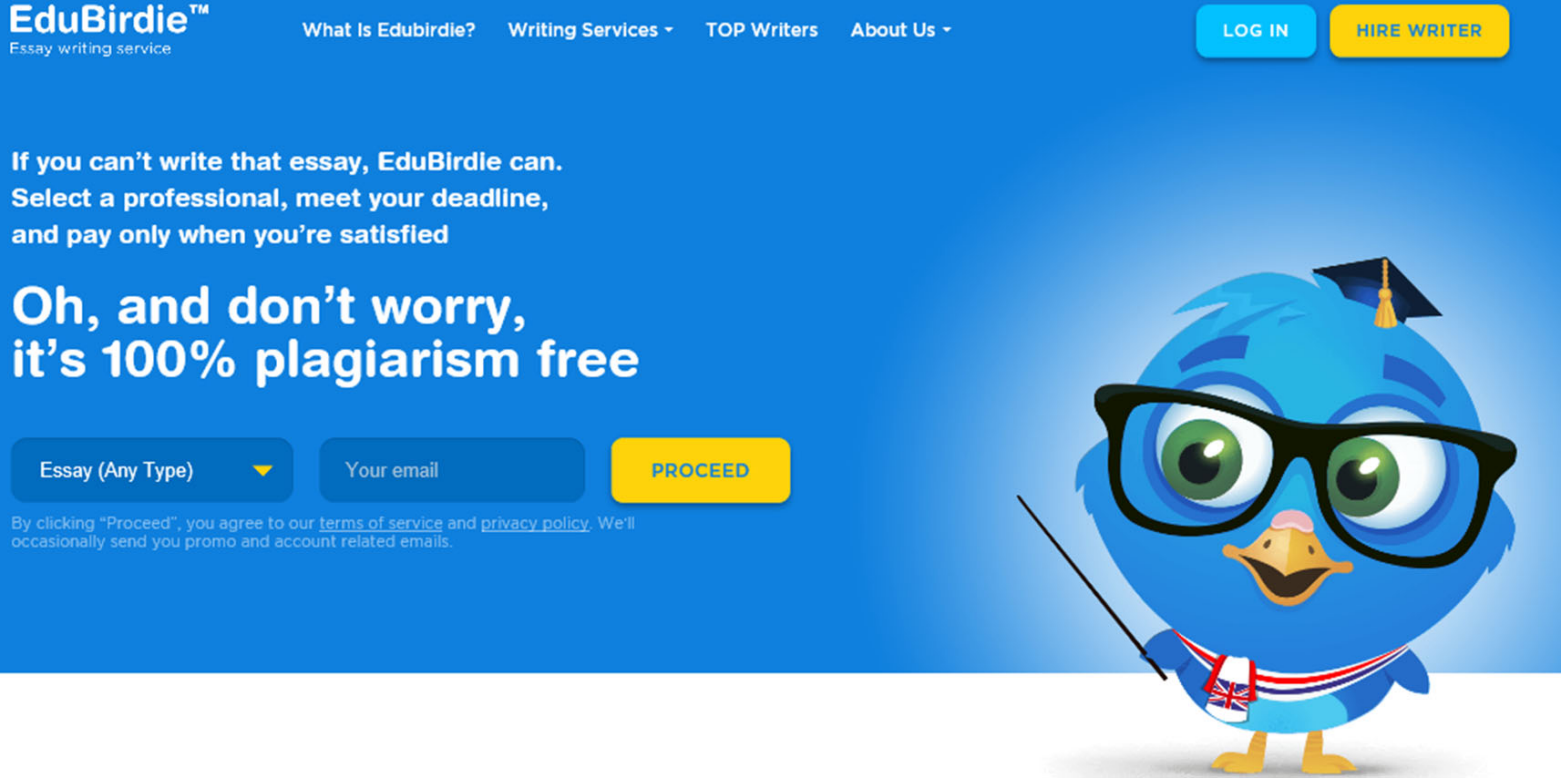
An example of an online advert offering a bespoke essay writing service to undergraduate students. ( Edubirdie.com, 2019)

Undergraduate medical education is susceptible to various forms of cheating ( Glick *et al*., 2001), with national and international examples documented ( Kusnoor *et al*., 2013; Tonkin 2015). This may be due to a combination of factors relating to both faculty and students, including a competitive culture in medical education (Kotter *et al*., 2017), the personality types that tend to be attracted to a career in medicine ( Eley, 2016) and the perceived lack of transparency by faculty in signposting what will be examined ( Tonkin 2015). The prevalence of cheating amongst medical students can be difficult to quantify, with estimates ranging between between 25% to 90% ( Henning *et al.,* 2013; Kusnoor *et al.,* 2013). Recently the issue has become increasingly sophisticated as digital platforms have provided an array of new technologies. An example of an online advert targeting students is shown in figure 1.

Cheating in higher education is reported to be rising ( Tonkin, 2015), with social media being used to compromise academic integrity within healthcare education ( Khadem-Rezaiyan *et al.,* 2017).

One case that received international attention involved students in Thailand sitting the Rangsit University Medical School entrance examination. Candidates wore glasses with inbuilt cameras to photograph the questions, which were transmitted via laptops during a break to an external source, who returned answers to the candidates’ smart watches ( Melvin, 2016). More recently, the University of Glasgow required their medical students to re-sit the end of year practical examination (objective structured clinical examination), following a small number of students using Facebook, WhatsApp and the University’s own messaging platform to share questions. This led to the medical school declaring the results void ( BBC, 2017). These institutions are not alone in these experiences; many medical schools have experienced similar issues ( Tonkin, 2015).

The effects of cheating can be significant. Technology can enable cheating to be achieved with accuracy, compromising the validity of the assessment process and placing into question an individual’s professionalism and probity. Such conduct may be a predictor of unprofessional behaviours in other areas, correlating with poor professional performance in the longer term, including within clinical environments which could impact upon both practitioners and patients ( Bertram *et al*., 2006).

Whilst there is discussion about ways to combat this, there is little yet published in the field. It is widely accepted to be a challenging area within higher education with responsibility placed on both students and faculty ( Gagnon *et al.,* 2015). Some authors argue that cheating is so firmly entrenched and normalised that attempts to tackle may prove futile ( Tonkin, 2015).

UCL Medical School has incorporated teaching on academic integrity in the digital age into its professionalism curriculum. Here, we present 12 tips on teaching academic integrity to medical students, which could equally be applied to other healthcare learners. We concentrate on an early introduction of an ‘academic integrity quiz’ which features real world scenarios. We do in addition cite principles learnt from other parts of the curriculum where we have incorporated academic integrity; namely professionalism, ethics and law sessions as well as relevant assessments.

## Practical tips on teaching academic digital integrity

### 1. ‘Prevention is better than cure’

Introducing key concepts to students early in their training can impact on subsequent behaviours ( Acharya *et al.,* 2017). Many undergraduate medical curricula adopt an integrated approach with early exposure to patients, clinical skills and aspects relevant to professionalism featured during the early years of the course. Since the consequences for students (and indeed doctors) of breaching professional codes can be costly and have significant personal, financial and academic implications, we introduce students to the issue of academic integrity in their first month at medical school. A systematic review highlighted that medical students who display such traits may also lack insight, and may benefit from teaching on issues such as academic integrity, in order to develop their self-reflection ( Vossen *et al.,* 2017). We therefore introduce students to the issue of academic integrity in their first month at medical school. Early introduction with dialogue between teachers and students can assist in highlighting those exhibiting potentially dubious attitudes, allowing educators to explore and manage underlying causes. A prophylactic approach is therefore logical in order to embed these elements of professionalism early.

### 2. Ensure there is professional role modelling by faculty

Issues of academic integrity is not limited to students; there have been reports of educators colluding with cheating ( Tonkin, 2015). Confusing messages from faculties as to what constitutes academic integrity may also complicate the issue. The lack of dialogue between students and staff on the topic has been highlighted as contributory to the problem ( Brockbank *et al.,* 2011). It is important that medical schools are clear in their standpoint on the matter as outlined by the governing bodies. The benefits of role modelling within education have influenced changes in the delivery of the medical curriculum, and ensuring all staff concur is likely to result positively on the uptake of the standards of professionalism expected amongst students ( Ellaway, 2013).

### 3. Adopt a non-punitive approach in teaching

It has been shown that students form lasting views on their medical school experience within the first few weeks of the course ( Nicholson, 2015). Whilst we introduce them early to academic integrity, we do not focus heavily at this point on possible disciplinary consequences. Whilst serious penalties for cheating do exist, using negative outcomes as a deterrent are not viewed as effective methods to change behaviour and may even serve as a distractor ( Tonkin, 2015); our aim is to allow students very early in their apprenticeships, to understand their professional responsibilities, potential sequelae of misdemeanours and the relevance to patients and the profession.

We do however emphasise the availability of support services available to students. Some of the underlying reasons for misdemeanours may arise from personal difficulties ( Yates *et al.,*2011). Adopting a holistic approach to the welfare of students and enhancing accessibility to support during higher education has been linked to better outcomes both academically and with regard to professional behaviours ( McLukie *et al.*, 2018). Like many medical schools, we assign a personal tutor throughout their medical school careers, as well as having regular contact with dedicated professionalism tutors.

### 4. Co-produce pedagogy with students

Acknowledging and fostering the expertise of students has been shown to enhance academic learning environments ( Armbruster *et al.,* 2009; Connell *et al.,* 2016). Collaboration with students who are ‘digital natives’ not only has potential to improve the accuracy of teaching materials but also to strengthen their ongoing professional learning ( Ellaway *et al.,* 2015). Involving students in co-creating their learning materials can have a variety of benefits. Co-production encourages students’ sense of educational responsibility as well as a heightened sense of trust and significance in the process, as highlighted by the concept of ‘powerful knowledge’ ( Harland, 2018). This can serve as a positive way to reinforce the standards of professionalism expected ( Rich, 2017). In co-authoring the academic integrity component of the course, collating experiences and opinions of our students have been central in development. Valuing students’ expertise and acknowledging the limitations of faculty are important in optimising relevance, accessibility and impact. In addition, patient and public engagement, especially with information technology experts and dedicated learning technologists, may improve pedagogic value ( Mayer, 2010; Sandars, 2012).

### 5. Use real examples to demonstrate the issues

Compromised academic integrity in the digital age can take many forms; from covert circulating of examination question banks and sharing of examination questions on online fora, to essay writing websites that ‘guarantee’ plagiarism-free material ( Fargen *et al.*, 2016). The situations and choices students may face are vast, with significant temptation. Our approach has been to contextualise the issue in the form of real world scenarios. National and local guidance describe what is expected in terms of professional behaviours, but do not always specifically name how these could present or appear. In our teaching materials, we introduce scenarios covering different forms of ‘assessment offences’ (plagiarism, self-plagiarism, contract cheating, collusion, fabrication and falsification) ( UCL Medical School, 2018). Scenario-based teaching has been demonstrated to improve student engagement, and promote problem solving skills relevant to all aspects of medicine ( Wood, 2003; Frost *et al.*, 2015; James *et al.,* 2015). Since many medical schools already feature ‘situational judgment test’ approaches (scenario based examination looking at decision making capabilities), many students are familiar with this pedagogy. By presenting realistic scenarios that students may encounter in a familiar format, we aim to increase relevance.Medical schools prepare graduating students for a challenging, complex workplace environment. This often involves providing them with a toolkit to recognise and negotiate factors including time management, stress and managing uncertainty. The scenarios that we use are therefore reflective of such factors and demonstrate the widespread potential repercussions of making unwise professional choices ( Leape *et al*., 2012; Yepes-Rios *et al.,* 2016).An example of a scenario used in teaching is as follows:

#### Scenario 1

Annie and Jamie are year 1 medical students. They are preparing for their Objective clinical and practical examinations (OCaPE). Annie is scheduled to undertake the examination tomorrow morning whilst Jamie is scheduled to undertake the examination tomorrow afternoon. Jamie suggests that Annie share with him via Snapchat the OCaPE stations after she completes the examination in the morning.

#### Please select the most appropriate action from the following options:

• Annie should comply with Jamie’s request, after all Jamie has been helpful with Annie’s preparations for the OCaPE examination. Collaboration is seen as a positive attribute in a future medical professional.
• Annie agrees to Snapchat Jamie the OCaPE stations but feels she does not want to disadvantage her friends who are also undertaking the examination in the afternoon. Annie agrees to Snapchat the OCaPE stations to Jamie and to the remainder of her friends.
• Annie feels uncomfortable with Jamie’s request. Annie states that she wants to fully focus on undertaking the OCaPE examination and does not feel she will be able to recall the content and will not comply with Jamie’s request.
• Annie feels uncomfortable with Jamie’s request, and does not agree to snapchatting him the examination stations as she feels this would be a form of cheating.
• I am uncertain as to which action would be most appropriate

### 6. Apply local and national guidance policy

The GMC’s Outcomes for Graduates ( 2018) outlines the standards of professionalism expected for newly qualifying doctors in the UK. Incorporation of regulatory guidance features throughout UK undergraduate medical schools.

In addition, universities and medical schools publish their own guidance as to what constitutes an assessment offence. Variations exist between policies with regard to terminology, penalties and how to escalate misdemeanours. It is therefore important to have current and consistent guidance and ensure that this is clearly signposted to students and faculty. In the quiz we use, students are asked to electronically submit what they feel constitutes the most appropriate action in a scenario. Following this, a concise explanation appears, with definitions of the type of assessment offence, linked to the relevant GMC guidance as well as medical school policy. We stress that this learning is formative only and does not contribute to a portfolio or academic progression.

For example, the explanation provided to scenario 1, above is provided as follows:

#### Correct answer

Annie feels uncomfortable with Jamie’s request, and does not agree to snapchatting him the examination stations as she feels this would be a form of cheating.

#### Explanation

If Annie Snapchats the examination stations to Jamie or anyone else this would be a form of ***collusion.***

Collusion is behaviour that may provide an advantage to another individual(s) during an assessment process. It is an assessment offence.

An assessment offence is defined as any sort of behaviour that results in unfair academic advantage.

Committing an assessment offence is defined as misconduct by UCL and will be investigated and students penalised.

The General Medical Council (GMC) provides guidance on professional behaviour for doctors. Even though you are training to become a doctor the GMC expects your level of professionalism to meet certain standards. This is a requirement needed to successfully graduate.

### 7. Educate the educators

To enable effective role modelling, ongoing training of the educators is vital to ensuring understanding of learning environments and evolving pedagogy. It is not uncommon for educators to lack familiarity with the latest influencing innovations in technology ( Stein *et al*., 2014). Utilising an interdisciplinary approach and upgrading the baseline knowledge of faculty opens opportunities for dialogue between educators as well as between educators and students. Lack of honest discussion has been highlighted as an inhibitor to tackling cheating ( Wrigley *et al.,* 2012). There is a school of thought amongst educationalists that the historic acceptance of cheating has made it part and parcel of medical culture ( Berlin, 2012; Ruhnke *et al.,* 2013). Educating our educators is one way to combat this using tutor sessions, INSET days, online learning modules, and sharing of local and national guidance.

### 8. Reinforce the message

Clinical reasoning abilities can develop at differing rates amongst learners. For example, reflective maturity tends to increase with age phronesis (clinical wisdom or intuition), whilst impulsivity tends to decrease ( O’Sullivan *et al.,* 2012). This emphasises the importance of revisiting these issues at multiple points during the medical school apprenticeship, increasing the chances of potential behaviour change ( O’Sullivan *et al.,* 2012)Transitioning into the later years of the course where the focus is on learning in a clinical environment, brings with it an array of additional experiences. No matter how contextualised learning material may be during the early years, reinforcing the topic by using principles of ‘spiral’ learning (which layers complexity as the learner progresses through the course) with educational content that mirrors their environment, can heighten professional relevance ( Byszewski *et al.,* 2015).

### 9. Adopt interactive methods to encourage dialogue

Didactic approaches to presenting professionalism have been found to be less impactful within healthcare education ( Dammers *et al.,* 2001; Dolmans *et al.,* 2005). Decision making is a key skill for medical students to acquire. Presenting students with real world scenarios may aid in developing decision making skills at an early stage ( Wood, 2003). We have used this position to construct scenarios in the form of a quiz; single best answers in a self-paced small group learning environment, making it clear that the results do not form a summative or formative assessment. Such models can be beneficial in encouraging dialogue and debate amongst students and exercise their early decision making skills ( Champaloux *et al*., 2016)An example of a scenario that we have used that particularly provoked debate amongst students and educators is illustrated below:

#### Scenario 2

Neil is a year 1 medical student. He has joined a study group. Adam is also in this study group. They are preparing for their formative written assessments. Adam shares a bank of past questions with the group. He obtained them from his older brother who graduated from the same medical school last year. Adam says that students would contribute to the bank yearly by attempting to remember as many questions as accurately as possible from the written examinations. The bank of questions would then be passed on to the year below. Adam says it is a “medical school tradition”.

**Please select the most appropriate action from the following options:**

• Neil is uncomfortable using the bank of questions and sees this as a form of cheating. He has benefited from the study group and does not wish to be ostracised. He declines Adam’s offer of using the questions but is happy to contribute to the bank of questions following the written assessment.
• Neil is happy to use and contribute to the bank of questions. He sees it as a privilege to be invited to partake in a “medical school tradition”.
• Neil is unhappy to use or contribute to the bank of questions. He feels this is a form of cheating and leaves the study group.
• Neil is happy to use the bank of questions. Neil is aware that many other students use similar banks of questions. Neil feels this “levels up the playing field” and does not see this as cheating.
• I am uncertain as to which action would be most appropriate

### 10. Continually review and update content

The digital world is constantly evolving, and curricular content should be updated accordingly. Contemporary content maintains relevance, resonates with students and is more likely to produce lasting results on the student cohort ( Goldenberg *et al*., 2018). An example of this includes a session we have introduced recently dedicated to social media and digital professionalism. This takes the form of a tutor led small group session focusing on the potential opportunities and pitfalls of social media with regards to confidentiality. Again, real world scenarios are extracted from Twitter, Instagram and Facebook and used as points of discussion on aspects of professionalism. By also acknowledging the positive aspects of modern technology, students are more likely to view digital opportunities as an adjunct rather than a short cut to their ongoing education ( Ko *et al.,* 2017). Regular student and faculty feedback should be obtained, quality assuring the learning materials used.

### 11. Integrate with other areas of the curriculum

Teaching academic integrity in the digital age may form one component of the undergraduate professionalism curriculum. Notably there are crossovers with many of the scenarios presented with areas including medical ethics, reflective practice, confidentiality and professional portfolios. Self-reflection in particular has been highlighted as a secondary outcome from the use of scenarios triggering conversations amongst students and educators. ( Rogers *et al.,* 2019). Incorporating elements of each within explanations to professional scenarios may provide students with an overall view of the importance of professionalism within their medical education. Integrating multiple curricula has been shown to further enhance the applicability and relevance of professionalism to clinical practice within medical education ( Foshee *et al.,* 2017).

### 12. Encourage lifelong professional learning and personal well-being

Academic integrity in the digital age is an issue that students face beyond their undergraduate careers into professional practice ( Gagnon, 2015). Evidence suggests that medical students who exhibit unprofessional behaviours are more likely to exhibit unprofessional behaviours later in their careers, in particular with regards to responsibility, initiative and self-improvement ( Fargen *et al.,* 2016). Incorporating contemporary issues within professionalism education at an early stage may positively impact on behaviours at later stages of medical careers.Evidence suggests that teaching on academic integrity not only translates into long term professionalism but can also provide an added health benefit in terms of maintaining a successful work-life balance ( Guthrie *et al.,* 1998). Within the UK, foundation year posts for qualifying junior doctors are allocated on a scoring system involving ranking based on academic performance ( GMC, 2018). Specialty doctor applications also involve professional portfolios, which include medical school experiences. Early academic performance can therefore contribute to future career paths and success. Incorporating discussion about academic integrity into the curriculum can highlight the pressures of competition amongst students and for some, uncover stressors which can contribute to burnout ( Guthrie *et al.,* 1998). In inviting these discussions, we may begin to address the issue of competition and burnout; the latter being a significant issue amongst doctors ( Gunasingam *et al.,* 2015). The intention is to therefore to start a conversation about lifelong professional well-being, as well as learning.

## Conclusions

Academic integrity is a significant issue within undergraduate medical education, which can have far reaching consequences for learners, patients and society. In teaching medical students about the importance and professional implications of academic integrity, our experience shows that an early, prophylactic approach that is co-created with students and based on real world situations encourages open dialogue between educators and students. The principles discussed in this 12 Tips article can be equally applied to other healthcare learners, both in undergraduate and postgraduate environments.

## Take Home Messages

Academic integrity is an important and challenging issue to tackle within the digital issue. Using a combination of methods including early prophylactic introduction, co-produced by students encourages open dialogue. The tips outlined can be equally applied to other learning envrionments both at undergraduate and postgraduate level.

## Notes On Contributors

Jenan Younis is a Colorectal surgeon and Education Fellow at UCL Medical School, London, UK ORCID: https://orcid.org/0000-0003-1771-6972

Faye Gishen is a consultant physician and Academic Lead for Clinical and Professional Practice at UCL Medical School, London, UK ORCID: https://orcid.org/0000-0003-2603-2759

## References

[ref1] Acharya Y. Rao M. Arja S. (2017) Evidence-based medicine in pre-clinical years: a study of early introduction and usefulness. BMC Medical Education. 17(164). PMC5522909 28761882

[ref2] Armbruster P. Patel M. Johnson E. Weiss M. (2009) Active learning and student-centered pedagogy improve student attitudes and performance in introductory biology. CBE Life Sciences Education. 8(3), pp.203–213. Peter Armbruster https://dx.doi.org/10.1187%2Fcbe.09-03-0025 19723815 10.1187/cbe.09-03-0025 PMC2736024

[ref3] BBC News , (2017) Glasgow medical students to resit exam after collusion. Available at: https://www.bbc.co.uk/news/uk-scotland-glasgow-west-39405833( Accessed: 01 June 2019)

[ref4] Berlin L. (2012) The ABR “recalls” conundrum: an ethical quandary. Journal of the American College of Radiology. 9, pp.380–383. https://doi.org/10.1016/j.jacr.2012.02.002 22632659 10.1016/j.jacr.2012.02.002

[ref5] Bertram T. Drinan P. (2006) Organizational theory and student cheating: explanation,responses, and strategies. Journal of Higher Education. 77, pp.839–860. https://doi.org/10.1080/00221546.2006.11778946

[ref6] Brockbank S. David T. Patel L. (2011) Unprofessional behaviour in medical students: a questionnaire-based pilot study comparing perceptions of the public with medical students and doctors. Medical Teacher. 33(9), pp.501–508. 10.3109/0142159X.2011.599450 21854145

[ref7] Byszewski A. Gill J. Lochnan H. (2015) Socialization to professionalism in medical schools: a Canadian experience. BMC Medical Education. 17(15), pp.204. 10.1186/s12909-015-0486-z PMC4650144 26577466

[ref8] Champaloux E. Keeley M. (2016) The impact of learning communities on interpersonal relationships among medical students. Medical Education. 1(21), pp.32958. 10.3402/meo.v21.32958 PMC5093321 27806828

[ref9] Connell G. Donovan D. Chambers T. (2016) Increasing the Use of Student-Centered Pedagogies from Moderate to High Improves Student Learning and Attitudes about Biology. CBE Life Sciences Education. 15(1). 10.1187/cbe.15-03-0062 PMC4803092 26865643

[ref10] Dammers J. Spencer J. Thomas M. (2001) Using real patients in problem-based learning: students’ comments on the value of using real, as opposed to paper cases, in a problem-based learning module in general practice. Medical Education. 35(1) pp.27–34. https://doi.org/10.1111/j.1365-2923.2001.00841.x 11123592 10.1046/j.1365-2923.2001.00841.x

[ref11] Dolmans D. De Grave W. Wolfhagen I. van der Vleuten C. (2005) Problem-based learning: future challenges for educational practice and research. Medical Education. 39(7), pp.732–741. https://doi.org/10.1111/j.1365-2929.2005.02205.x 15960794 10.1111/j.1365-2929.2005.02205.x

[ref12] Edubirdie , (2018) Essay writing service. Available at: https://uk.edubirdie.com( Accessed: 01 June 2019)

[ref13] Eley D. Leung J. Hong B. Cloninger K. (2016) Identifying the Dominant Personality Profiles in Medical Students: Implications for Their Well-Being and Resilience. PLoS One. 5(8). 10.1371/journal.pone.0160028 PMC4975484 27494401

[ref14] Ellaway R. (2013) Cheating and the new moral compass. Medical Teacher. 35(6), pp.526–528. https://doi.org/10.3109/0142159X.2013.808923 23705656 10.3109/0142159X.2013.808923

[ref15] Fargen K. Drolet B. Philibert I. (2016) UnprofessionalBehaviors Among Tomorrow’s Physicians: Review of the Literature With a Focus on Risk Factors, Temporal Trends, and Future Directions. Academic Medicine. 91(6), pp.858–864. 10.1097/ACM.0000000000001133 26910897

[ref16] Foshee C. Mehdi A. Bierer S. Traboulsi E. (2017) A Professionalism Curricular Model to Promote Transformative Learning Among Residents. The Journal of Graduate Medical Education. 9(3), pp.351–356. 10.4300/JGME-D-16-00421.1 28638516 PMC5476387

[ref17] Frost K. Metcalf E. Brooks R. Kinnersley P. (2015) Teaching pediatric communication skills to medical students. Advances in Medical Education and Practice. 6, pp.35–43. https://doi.org/10.2147/AMEP.S68413 25653569 10.2147/AMEP.S68413 PMC4303365

[ref18] Gagnon K. and Sabus C. (2015) Professionalism in a digital age: opportunities and considerations for using social media in health care. Physical Therapy. 95(3), pp.406–414. 10.2522/ptj.20130227 24903111

[ref19] General Medical Council, (2018) Available at: https://www.gmc-uk.org/registration-and-licensing(Accessed: 01 June 2019)

[ref20] General Medical Council , (2018) Outcomes for Graduates. Available at: https://www.gmc-uk.org/education/standards-guidance-and-curricula/standards-and-outcomes/outcomes-for-graduates( Accessed: 01 June 2019)

[ref21] Glick S. (2001) Cheating at medical school. BMJ. 1, pp.322. http://dx.doi.org/10.1136/bmj.322.7281.250 10.1136/bmj.322.7281.250 PMC1119511 11157511

[ref22] Goldenberg M. Lee J. (2018) Surgical Education, Simulation, and Simulators-Updating the Concept of Validity. Current Urology Reports. 19(7), pp.52. 10.1007/s11934-018-0799-7 29774439

[ref23] Gunasingam N. Burns K. Edwards J. Dinh M. (2015) Reducing stress and burnout in junior doctors: the impact of debriefing sessions. Postgraduate Medical Journal. 91(1074), pp.182–187. 10.1136/postgradmedj-2014-132847 25755266

[ref24] Guthrie E. Black D. Bagalkote H. Shaw C. (1998) Psychological stress and burnout in medical students: a five-year prospective longitudinal study. Journal of the Royal Society of Medicine. 91(5), pp.237–243. 10.1177/014107689809100502 9764076 PMC1296698

[ref25] Henning M. Ram S. Malpas P. Shulruf B. (2013) Academic dishonesty and ethical reasoning: pharmacy and medical school students in New Zealand. Medical Teacher. 35, pp.1211–1217. 10.3109/0142159X.2012.737962 23146078

[ref26] James H. Khalid A. Sequeira R. (2015) Effective use of real-life events as tools for teaching-learning clinical pharmacology in a problem-based learning curriculum. Indian Journal of Pharmacology. 47(3), pp.316–321. https://dx.doi.org/10.4103%2F0253-7613.157131 26069371 10.4103/0253-7613.157131 PMC4450559

[ref27] Khadem-Rezaiyan M. and Dadgarmoghaddam M. (2017); Research Misconduct: A Report from a Developing Country. Iranian Journal of Public Health. 46(10), pp.1374–1378. 10.1007/s11948-016-9767-0 29308381 PMC5750349

[ref28] Ko L. Rana J. Burgin S. (2017) Incorporating social media into dermatologic education. Dermatology Online Journal. 23(10). 29469778

[ref29] Kötter T. Wagner J. Brüheim L. Voltmer E. (2017) Perceived Medical School stress of undergraduate medical students predicts academic performance: an observational study. BMC Medical Education. 17(1). 10.1186/s12909-017-1091-0 PMC5732510 29246231

[ref30] Kusnoor A. and Falik R. (2013) Cheating in medical school: the unacknowledged ailment. Southern Medical Journal. 106, pp.479–483. 10.1097/SMJ.0b013e3182a14388 23912144

[ref31] Harland T. and Wald N. (2018) Curriculum, teaching and powerful knowledge. Higher Education. 228. 10.1007/s10734-017-0228-8

[ref32] Leape L. Shore M. Dienstag J. Mayer R. (2012) Perspective: a culture of respect, part 2: creating a culture of respect. Academic Medicine. 87(7), pp.853–858. 10.1097/ACM.0b013e3182583536 22622219

[ref33] Mak-van der Vossen M. van Mook W. van der Burgt S. Kors J. (2017) Descriptors for unprofessional behaviours of medical students: a systematic review and categorisation. BMC Medical Education. 17(1). https://dx.doi.org/10.1186%2Fs12909-017-0997-x 10.1186/s12909-017-0997-x PMC5603020 28915870

[ref34] Mayer R. (2010) Applying the science of learning to medical education. Medical Education. 44(6), pp.543–549. 10.1111/j.1365-2923.2010.03624.x 20604850

[ref35] McLukie A. Matheson K. Landers A. Landine J. (2014) What dental educators need to understand about emerging technologies to incorporate them effectively into the educational process. Journal of Dental Education. 78(4), pp.520–529. 24706681

[ref36] Melvin D. Students snared in high tech cheating scam in Thailand.(2016) CNN. https://edition.cnn.com/2016/05/11/asia/thailand-high-tech-examination-cheating/index.html

[ref37] Nicholson S. (2018) The Relationship Between Psychological Distress and Perception of Emotional Support in Medical Students and Residents and Implications for Educational Institutions. Academic Psychiatry. 42(1), pp.41–47. 10.1007/s40596-017-0800-7 29124715

[ref38] O’Sullivan H. van Mook W. Fewtrell R. Wass V. (2012) Integrating professionalism into the curriculum. Medical Teacher. 34(2), pp.155–157. 10.3109/0142159X.2012.655610 22288994

[ref39] Rich J. (2017) Proposing a Model of Co-Regulated Learning for Graduate Medical Education. Academic Medicine. 92(8), pp.1100–1104. 10.1097/ACM.0000000000001583 28177957

[ref40] Rogers S. Priddis L. Michels N. Tieman M. (2019) Applications of the reflective practice questionnaire in medical education. BMC Medical Education. 19(1). https://doi.org/10.1186/s12909-019-1481-6 10.1186/s12909-019-1481-6 PMC6367754 30732611

[ref41] Ruhnke G. and Doukas D. (2013) Trust in residents and Board examinations: when sharing crosses the boundary. Mayo Clinic Proceedings. 88, pp.438–441. 10.1016/j.mayocp.2013.02.003 23639496 PMC4862587

[ref42] Sandars J. (2012) Technology and the delivery of the curriculum of the future: opportunities and challenges. Medical Teacher. 34(7), pp.534–538. 10.3109/0142159X.2012.671560 22746960

[ref43] Stein C. Eisenberg E. O’Donnell J. Spallek H. (2014) What dental educators need to understand about emerging technologies to incorporate them effectively into the educational process. Journal of Dental Education. 78(4), pp.520–529. 24706681

[ref44] Tonkin A. (2015) Lifting the carpet on cheating in medical school exams. BMJ. 18(351). https://doi.org/10.1136/bmj.h4014 10.1136/bmj.h4014 26286467

[ref45] UCL Medical School (2018) Policies and regulations. Available at: https://www.ucl.ac.uk/medical-school/current-mbbs-students/general-information/policies-and-regulations( Accessed: 01 June 2019)

[ref46] Wood D. (2003) Problem based learning. BMJ. 326(7384), pp.328–330. https://dx.doi.org/10.1136%2Fbmj.326.7384.328 12574050 10.1136/bmj.326.7384.328 PMC1125189

[ref47] Wrigley W. Vleuten C. Freeman A. Muijtjens A. (2012) A systemic framework for the progress test: strengths, constraints and issues. Medical Teacher. 34(9), pp.683–697. 10.3109/0142159X.2012.704437 22905655

[ref48] Yates J. (2011) Development of a toolkit to identify medical students at risk of failure to thrive on the course: an exploratory retrospective case study. BMC Medical Education. 18(11). 10.1186/1472-6920-11-95 PMC3229499 22098629

[ref49] Yepes-Rios M. Dudek N. Duboyce R. Curtis J. (2016) The failure to fail underperforming trainees in health professions education: A BEME systematic review. Medical Teacher. 38(11), pp.1092–1099. https://doi.org/10.1080/0142159X.2016.1215414 27602533 10.1080/0142159X.2016.1215414

