# Supplementary material for: Practical tips for teaching academic integrity in the digital age
Source: MedEdPublish (2016). 2019 Jun 21;8:142. [Version 1] doi: 10.15694/mep.2019.000142.1 (PMC10712489; doi:10.15694/mep.2019.000142.1)
Supplement: Peer review history

# Reviewer report: Reviewer response for version 1

**DOI:** 10.21956/mep.19817.r29846

**Published:** 2019-07-30

**Authors:** Jayne Garner

**Affiliations:** 1 university of liverpool

This review has been migrated. The reviewer awarded 4 stars out of 5 These tips provide good background evidence regarding integrity, and some practical examples/scenarios which could be included in discussions and personal professional development sessions with students. This is a valuable addition to a challenging issue - thank you.

Reviewer Expertise:

NA

No decision status is available

# Reviewer report: Reviewer response for version 1

**DOI:** 10.21956/mep.19817.r29845

**Published:** 2019-06-23

**Authors:** Felix Silwimba

**Affiliations:** 1 University of Lusaka

This review has been migrated. The reviewer awarded 5 stars out of 5 I will term this article as excellent, especially for the first world educators and learners. it is still relevant for learners in LIMC countries even though they are some limitations in digital advancement. I would in addition suggest that the recommendations are extended to high school learners because that is where the damage is caused.

Reviewer Expertise:

NA

No decision status is available

# Reviewer report: Reviewer response for version 1

**DOI:** 10.21956/mep.19817.r29844

**Published:** 2019-06-21

**Authors:** Tan Nguyen

**Affiliations:** 1 Deakin University

This review has been migrated. The reviewer awarded 5 stars out of 5 I thoroughly enjoyed reading this paper. The authors provided practical examples and referred to the relevant and recent literature in the subject field.

Reviewer Expertise:

NA

No decision status is available
